# Short-lasting unilateral neuralgiform headache attacks with cranial autonomic symptoms (SUNA) secondary to epidermoid cyst in the right cerebellopontine angle successfully treated with surgery

**DOI:** 10.1007/s10194-011-0326-4

**Published:** 2011-03-16

**Authors:** Pedro Enrique Jiménez Caballero, Juan Carlos Portilla Cuenca, Ignacio Casado Naranjo

**Affiliations:** 1 Department of Neurology, San Pedro de Alcántara Hospital, Avenida Pablo Naranjo, 10002 Cáceres, Spain; 2 Calle Dionisio Acedo no 9, Portal 7, 4-1a, 10001 Cáceres, Spain

**Keywords:** SUNA, Epidermoid cyst, Cerebellopontine angle, Surgery, Treatment

## Abstract

Short-lasting unilateral neuralgiform headache attacks with conjunctival injection and tearing (SUNCT) syndrome is a rare headache syndrome classified among the trigeminal autonomic cephalalgias. It is usually idiopathic, although infrequent secondary forms have been described. Recently, the term short-lasting unilateral headache with cranial autonomic symptoms (SUNA) has been defined by the International Headache Society (ICHD-2) as similar to SUNCT with less prominent absent conjunctival injection and lacrimation. We report a patient with paroxysmal orbito-temporal pains, phenotypically suggesting SUNA, secondary to epidermoid cyst in the cerebellopontine angle which disappeared after tumor resection. Neuroimaging should be considered in all patients with SUNA, notably in those with atypical presentation as our patient who presented on examination trigeminal hypoesthesia and tinnitus. Realization of a brain MRI would rule out injuries that causes this type of syndrome.

## Introduction

Short-lasting unilateral neuralgiform headache with conjunctival injection and tearing (SUNCT) is a rare form of trigeminal autonomic cephalalgia (TAC) mostly described as a primary headache syndrome. The acute headache is usually accompanied by ipsilateral conjunctival injection, lacrimation, rhinorrhoea, nasal congestion, ptosis, miosis and facial redness or sweating. Short-lasting unilateral neuralgiform headache with autonomic symptoms (SUNA) has been defined by the International Headache Society (ICHD-2) as similar to SUNCT with less prominent or absent conjunctival injection and lacrimation [1]. SUNCT syndrome has been described secondary to other causes [2]. Posterior fossa lesions, including ipsilateral cerebellopontine angle arteriovenous malformations, brainstem cavernous haemangioma and base of skull bony abnormalities have been described anecdotally to be associated with SUNCT.

## Case report

The patient was a 36-year-old woman with pain from a deep area of the right eye to the temporal region as the primary symptom. In August 2009, attacks of pulsating and stabbing pain in the right orbital to temporal region with a sudden onset and duration of about 30 s began to occur 8–12 times almost daily. It did not occur more frequently during any particular period of the day. She denied having any triggers for these attacks. Photophobia and phonophobia were noted during the attacks, but there was no nausea, vomiting, conjunctival injection, or tearing. Nasal congestion was the only autonomic symptom. The patient was prescribed carbamazepine and ibuprofen, but there were no changes in the symptoms. The patient also consulted an ophthalmologist, but there was no evidence ocular pathology.

**Fig. 1:**
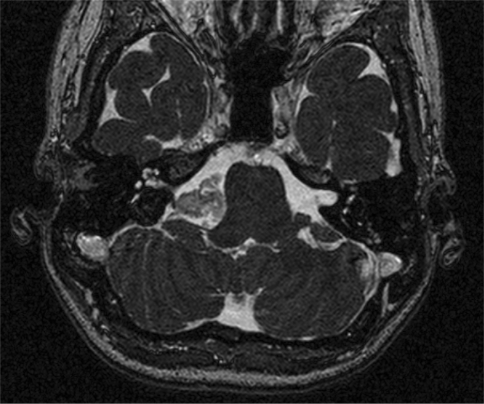
Brain MRI in axial plane and T2 sequences: extraaxial lesion at the right cerebellopontine angle, heterogeneous, hyperintense with edges well defined. The lesion produces imprint on the brain stem without signs of infiltration. All these features are suggestive of an epidermoid cyst

She visited the outpatient clinic of our department in the late October 2009. She was previously healthy and had no familial history of migraine. The examination was performed in an attack-free period, no swelling or reddening of the scalp arteries including the superficial temporal artery was noted, and no herpetic rash was observed on her face or head. No particular neurological abnormality was noted, except a hypoesthesia in the first and second right trigeminal branches territories and tinnitus. Blood test showed no abnormality in peripheral blood or general biochemical analysis, no sign of inflammatory reaction, and no abnormality related to collagen disease or any hypothalamohypophyseal hormones such as prolactin or thyroid hormones. Treatment was changed to gabapentin (1,600 mg/day) and indomethacin (75 mg/day) without improvement. Brain MRI (Fig. 1) revealed an extraaxial lesion at the right cerebellopontine angle with edges well defined, hypointense on T1 (relative to the cerebral cortex), hyperintense on T2, heterogeneous in FLAIR sequences, which does not capture contrast. The lesion displaces the VII and VIII cranial nerve, and produces imprint on the brain stem and adjacent right cerebellar hemisphere, without signs of infiltration. All these features are suggestive of an epidermoid cyst.

A total resection of the lesion was carried out by retrosigmoid craniectomy without complications. During surgery no evidence of any vascular loop on the trigeminal nerve was observed. SUNA episodes gradually disappeared in less than a week. When the patient was asymptomatic the gabapentin was stopped. In reviews at 6 and 12 months she continued asymptomatic.

## Discussion

TACs are a group of primary headache disorders characterized by unilateral pain in the trigeminal nerve distribution, associated with ipsilateral cranial autonomic features. Despite their similarities, these disorders differ in their clinical manifestations and response to therapy. We excluded paroxysmal hemicrania based on the lack of response to indomethacin [3]. Additionally, the patient does not have trigger points or response to carbamazepine, and the pain duration is longer than that usually observed in trigeminal neuralgia [4]. Primary idiopathic stabbing headache is another differential diagnosis we considered. However, in 80% of cases with this diagnosis, the pain lasted less than 2 s, and in 40% of the cases, patients report other migraine features. Additionally, the pain is usually (but not always) responsive to indomethacin and cranial autonomic features are absent in this syndrome [5]. In view of the absence of conjunctival injection or tearing, our patient satisfies the definition of SUNA (Table 1).

**Table 1:** Classification of SUNA according to the second edition of the International Classification of Headache Disorders

A At least 20 attacks fulfilling criteria B–E
B Attacks of unilateral orbital, supraorbital or temporal stabbing or pulsating pain lasting from 2 s to 10 min
C Pain is accompanied by one of
1. Conjunctival injection and/or lacrimation
2. Nasal congestion and/or rhinorrhea
3. Eyelid oedema
D Attacks occur with a frequency of ≥1 per day from more than half of the time
E No refractory period follows attacks triggered from trigger areas
F Not attributed to another disorder

Recently, criteria have been proposed to establish a causal relationship between the associated lesion and the headache syndrome [6] (Table 2). Our patient satisfied all of them. Even clinically typical TACs can be caused by structural lesions. Neuroimaging should be considered in all patients with TACs or TAC-like syndromes, notably in those with atypical presentation [7] as our patient who presented on examination trigeminal hypoesthesia and tinnitus.

**Table 2:** Criteria for determining causal relationship between associated pathology and headache

1.	Close temporal relationship between the associated disease and the onset of pain
2.	Side concordance between the unilateral pain and the lesion, if localized
3.	Surgical remission, if the patient was operated on, or prompt remission after aetiological medical therapy, if indicated, without the need of constant indomethacin administration
4.	Prolonged post-treatment follow-up, in order to exclude a relapse of the headache attacks or improvement due to spontaneous remission

Most cases of SUNCT are idiopathic, but there are a few cases in the literature that are secondary to intracranial lesions. They are either due to pituitary lesions or posterior fossa lesions [8] as vascular compression of trigeminal nerve. However, symptomatic SUNA cases are rare in the literature. They have been described secondary to multiple sclerosis [9], vertebral artery dissection, cortical dysplasia and post-traumatic. In a study [9], there were no SUNA patients whose attacks could be triggered by touch, as opposed to 63% of SUNCT patients in whom touching the face could trigger attacks. These authors reported that uni or bilateral activation of the hypothalamus was noted during headache attacks in patients with SUNCT, but that no activation of the same regions was noted in those with SUNA. Microvascular decompression of the trigeminal nerve in medically intractable SUNCT and SUNA in patients that demonstrate aberrant arterial loop imprint could be effective [10].

To our knowledge, this patient is the first case of SUNA secondary to epidermoid cyst and causality is strengthened by disappearance of episodes of pain after tumor resection.

## References

[1] The International Classification of Headache Disorders (2004) Headache subcommittee of the International Headache Society. Cephalalgia 24(Suppl 1):1–50 10.1111/j.1468-2982.2003.00824.x 14979299

[2] Kutschenko A, Liebetanz D (2010). Meningioma causing gabapentin-responsive secondary SUNCT syndrome. J Headache Pain.

[3] Price RW, Posner JB (1978). Chronic paroxysmal hemicrania: a disabling headache responding to indomethacin. Ann Neurol.

[4] Consentino G, Fierro B, Puma AR, Talamanca S, Brighina F (2010). Different forms of trigeminal autonomic cephalalgias in the same patient: description of a case. J Headache Pain.

[5] Pareja JA, Kruszewski P, Caminero AB (1999). SUNCT syndrome versus idiopathic stabbing headache (jabs and jolts syndrome). Cephalalgia.

[6] Trucco M, Mainardi F, Maggioni F, Badino R, Zacharin G (2004). Chronic paroxysmal hemicrania, hemicrania continua and SUNCT syndrome in association with other pathologies: a review. Cephalalgia.

[7] Wilbrink LA, Ferrari MD, Kruit MC, Haan J (2009). Neuroimaging in trigeminal autonomic cephalalgias: when, how and what. Curr Opin Neurol.

[8] Adamo MA, Dranzin D, Popp AJ (2008). Short-lasting unilateral neuralgiform headache attacks with conjunctival injection and tearing syndrome treated successfully with transsphenoidal resection of a growth hormone-secreting pituitary adenoma. J Neurosurg.

[9] Cohen AS, Matharu MS, Goadsby PJ (2006). Short-lasting unilateral neuralgiform headache attacks with conjunctival injection and tearing (SUNCT) or cranial autonomic features (SUNA). A prospective clinical study of SUNCT and SUNA. Brain.

[10] Williams M, Bazina R, Tan L, Rice H, Broadley SA (2010). Microvascular decompression of the trigeminal nerve in the treatment of SUNCT and SUNA. J Neurol Neurosurg Psychiatry.

